# Oil Spill Sorber Based on Extrinsically Magnetizable Porous Geopolymer

**DOI:** 10.3390/ma14195641

**Published:** 2021-09-28

**Authors:** Fabíola da Silveira Maranhão, Fernando Gomes, Sérgio Thode, Diganta B. Das, Emiliane Pereira, Nathali Lima, Fernanda Carvalho, Mostafa Aboelkheir, Vitor Costa, Kaushik Pal

**Affiliations:** 1 Instituto de Macromoléculas, Professora Eloisa Mano, Centro de Tecnologia-Cidade Universitária, Av. Horacio Macedo, 2030, Bloco J. Universidade Federal de Rio de Janeiro, Rio de Janeiro 21941-598, RJ, Brazil; fabiola.smaa@gmail.com (F.d.S.M.); emilianedaher@gmail.com (E.P.); nathali.rbl@gmail.com (N.L.); fernandaveloso.carvalho@yahoo.com.br (F.C.); vitor.cdc94@gmail.com (V.C.); kaushikphysics@gmail.com (K.P.); 2 Programa de Engenharia da Nanotecnologia, COPPE, Centro de Tecnologia-Cidade Universitária, Av. Horacio Macedo, 2030, Bloco I. Universidade Federal do Rio de Janeiro, Rio de Janeiro 21941-594, RJ, Brazil; 3 Núcleo de Monitoramento Ambiental, Instituto Federal de Ciência e Tecnologia do Rio de Janeiro, Av. República do Paraguai, 120, Vila Sarapui, Duque de Caxias 25050-100, RJ, Brazil; sergio.thode@ifrj.edu.br; 4 Department of Chemical Engineering, Loughborough University, Loughborough LE113TU, Leicestershire, UK; D.B.Das@lboro.ac.uk; 5 Programa de Engenharia Civil, Universidade São Judas Tadeu, Rua Taquari, 546, Mooca, São Paulo 03166-000, SP, Brazil; prof.mostafamohamed@usjt.br

**Keywords:** magnetic geopolymer, light materials, environmental recovery, oil, magnetic nanoparticles

## Abstract

Environmental impacts are increasingly due to the human polluting activities. Therefore, there is a need to develop technologies capable of removing contamination and driving the impacted environment as close as possible to its inherent characteristics. One of the major problems still faced is the spill of oil into water. Therefore, to solve the environmental problem, this work shows the use of magnetically modified geopolymer materials as an oil remover from water with a magnet’s aid. The results obtained were outstanding since the average intrinsic oil removal capability (IORC) was 150 g/g. The presented IORC is the largest found in the materials produced by our research group, constituting an extremely encouraging result, mainly because of the ease of preparing the magnetic geopolymer system. Furthermore, the low cost of production and the material’s capability to be reused as filler of polymer or even cementitious matrices allows us to project that this nanocomposite can be widely used, constituting an economically viable alternative for more efficient environmental recovery processes.

## 1. Introduction

Oil and gas are chemical compounds of great industrial, economic, and social potential capable of bringing benefits to life quality since most of the energy and materials currently obtained come from the different oil and gas processing stages. However, the extraction of these goods is still a challenge in preserving environmental quality, as many accidents, such as oil spills at sea, still occur. Oil spill accidents, such as that in the Mexico Gulf, produce severe damage to the ecosystem due to the rapid dispersion of the oil in the sea [1].

Large amounts of oil were released in the sea of Paraíba, Brazil, in 2020. The oil spread over more than 70% of the 3300 km of northeastern Brazilian states, reaching almost 500 sites in early November [2]. Given this scenario, technologies involving chemical and biological treatments must be developed continuously. Chemical treatments are those aimed at producing compounds capable of facilitating the burning, sorption, or dispersion of the oil [3].

In this context, geopolymers are promising materials. Geopolymers are classified as inorganic polymers, as their composition includes repeated units of aluminosilicates that form a three-dimensional structure with covalent bonds. The reaction occurs from the mixture of the aluminosilicate with an alkaline solution (sodium hydroxide and sodium silicate) responsible for activating the reaction [4]. The cost of the production of geopolymers is low since it can be made from residual raw material rich in aluminosilicates, such as: fly ash, ash obtained from burning rice husks, red mud, glass powders, sedimentary rocks, clays, and metakaolin [5].

Geopolymer sorption capability can be improved by the insertion of H_2_O_2_, which confers porosity to the matrix. Moreover, the use of magnetic fillers can also improve the sorption capability of these matrices. Magnetic nanoparticles are often used in application fields such as absorption, separation, sensing, and electromagnetic dissipation [6,7,8,9,10,11]. Ordinarily, geopolymers have been used to recover environments degraded by heavy metals [12]. Sorption of these metallic species occurs due to the positive and negative charges present in the geopolymer structure, composed of aluminum, silica, oxygen, and sodium as a counter-ion [13]. The oil has different chemical compounds, with different electrical charges and polarizability. Thus, the ions and cations in the geopolymer matrix can attract and trap the oil.

Therefore, this work proposed using magnetic geopolymers to perform oil spill clean-up operations under a magnetic field. The results showed that one gram of the material removes 67 g of the petroleum from the water. Our research group has been working for several years with macromolecular systems capable of removing oil from water [14,15,16,17,18,19,20,21,22,23,24,25,26]

Among these macromolecules, phenolic resins based on cashew nut liquid and cardanol/furfural have already been tested. Polyurethane systems based on castor oil have also been tested, and we have also tested polyester poly (butylene succinate). One gram of the best ones among these materials can remove 11 g [23] and 10 g [22] of oil from the water, respectively. Sorption tests proved that the materials produced here presented an average intrinsic oil removal capability of around 150 g/g. This outstanding response, coupled with the low cost of the material and its ease of preparation, allows us to consider that the geopolymer matrices are potent allies in environmental recovery processes.

## 2. Materials and Methods

### 2.1. Synthesis of Magnetic Nanoparticles Modified with SiO_2_ and the Production of Composites

The synthesis of the magnetic nanoparticles modified with SiO_2_ consisted of modifying the magnetic particles with silica so that there was a more significant geopolymer/magnetite interaction. Thus, iron salts (Fe^2+^ and Fe^3+^) at 0.46 M and a solution of SiO_2_ and NaOH at 4.33 M were prepared. The latter was then added to the iron salts solution and left to stir for 2 h at 300 rpm until the nanoparticles ultimately precipitated. Finally, the samples were decanted with a magnet’s aid, washed three times with distilled water, and placed in the oven at 50 °C to dry.

The geopolymers were synthesized with the modified magnetic particles. For this purpose, an alkaline solution (NaOH) was prepared at 12 M and left to stir for 1 min, then metakaolin was added to the alkaline solution in the molar ratio of Na_2_O/Al_2_O_3_ = 1. The pore-forming agent (H_2_O_2_) at 1.5% w/w and the modified magnetic nanoparticles in concentrations of 1%, 2%, and 3% were added to each synthesis performed and left stirring for five minutes at 300 rpm. Finally, the composites formed were taken to the oven for curing at 80 °C for two days.

### 2.2. Characterizations

#### 2.2.1. X-ray Diffraction (XRD)

X-ray diffraction measurements were performed using a device SHIMADZU model DRX-6000 (SHIMADZU, Kyoto, Japan), located at the Instituto de Macromoléculas Professora Eloisa Mano, Rio de Janeiro, Brazil. In normal temperature and atmospheric pressure conditions, the equipment works with a copper source (Cu Kα = 0.154 nm) under 40 kV and 20 mA. The crystalline size (Lc) was calculated using the Scherrer’s Equation [27] (Equation (1)).

(1) $$\frac{\mathrm{Kx}\lambda }{\beta \mathrm{xcos}\theta }$$

where Lc is the crystalline size, λ is the wavelength, β is the half-width, and *θ* is the diffraction angle.

#### 2.2.2. Fourier Transform Infrared Spectroscopy with Attenuated Total Reflectance Accessory (FTIR–ATR)

FTIR–ATR analyses of the samples’ powder were performed in a Perkin-Elmer 1720X Fourier transform spectrometer (Perkin-Elmer, Waltham, MA, USA), located at the Instituto de Macromoléculas Professora Eloisa Mano, Rio de Janeiro, Brazil..The FTIR spectra were obtained using ATR (diamond crystal) in an inert atmosphere, with a resolution of 4 cm^−1^ in the range 4000–675 cm^−1^. Stored results were averages of 124 scans.

#### 2.2.3. Scanning Electron Microscopy (SEM) and Transmission Electron Microscopy (TEM)

SEM analyses were performed using JEOL JSL 5300 Microscope Instruments (JEOL, Tokyo, Japan), operating at five keV, set to use the secondary electron back-scattering electron detectors, located at the Mineral Technology Center, Rio de Janeiro, Brazil. The software ImageJ was used for the treatment of micrographs and the determination of the particle sizes. In turn, samples were prepared for TEM analysis by drying nanoparticles on a copper grid that is coated with a thin layer of carbon. Then they were examined under a FEI-Tecnai Spirit 12 Transmission Electron Microscope, United States, USA, located at the COPPE, Federal University of Rio de Janeiro, Rio de Janeiro, Brazil.

#### 2.2.4. Porosity Analyses

The porosity test was performed using a 25 mL beaker, where 0.45 g of geopolymer was added. Then, water was added until the saturation of the geopolymer matrix. The volume was used to perform the calculations [28], following Equation (2):

(2) $$Pt=\frac{Pv}{Vt}$$

where *Pt* is the Porosity, *Pv* is the Pore volume, and *Vt* is the Total volume.

#### 2.2.5. Magnetic Force Test

**Figure 1:**
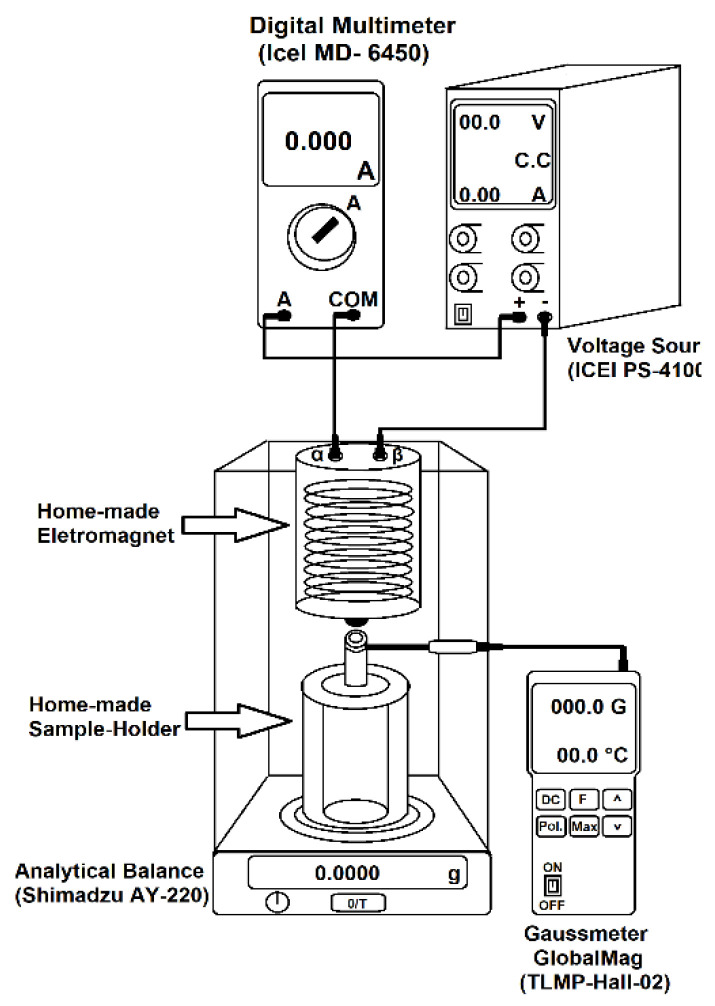
Magnetic force test scheme.

Magnetic force tests were performed using a homemade experimental setup, described elsewhere. This setup is constituted by an analytical balance Shimadzu (Kyoto, Japan) AY-220, a voltage source ICEL PS-4100, a digital multimeter ICEL MD-6450, a gaussmeter GlobalMag TLMP-Hall-02, a homemade sample holder; and a homemade electromagnet, located at the Instituto de Macromoléculas Professora Eloisa Mano, Rio de Janeiro, Brazil. System calibration was performed in the absence of magnetic material. Firstly, using the amperemeter and the gaussmeter, a current versus magnetic field calibration was performed. Afterward, a current versus mass calibration was also performed. Obtained results were used to predict part of the presented error. Magnetic force tests were performed following the mass variation of the sample in the magnetic field’s presence, produced by the electromagnet. Then, the apparent variation of mass of the sample in the presence of the magnetic field was calculated by subtracting the sample’s mass in the magnetic field’s presence from the sample’s mass. The magnetic force (opposite to gravitational one) was calculated according to Equation (3):

(3) $$Fm=\frac{\Delta \mathrm{mxg}}{\begin{array}{c} m0\;\; \end{array}}$$

where *Fmn* is the magnetic force normalized by the initial mass of the sample *m*0, Δ*m* is the apparent variation of mass in the magnetic field’s presence, and *g* is the acceleration of gravity. The Figure 1 shows the magnetic force test.

#### 2.2.6. Crude Oil Magnetic Removal

These tests were performed at room temperature using synthetic brine. The brine was prepared using 29.234 g sodium chloride and 0.406 g calcium chloride [29]. The crude oil used presented a density equal to 0.9730 g/mL and 13°API (@60 °F). The oil emulsion was prepared using the methodology of WANG and collaborators, with some modifications, so that the dispersion of the oil in water was efficient, as it was done at room temperature at 30 °C [30]. In this sense, a 100 mL beaker containing 90 mL of brine was used, into which 0.5 g of crude oil was spilled. After that, a known weight of the absorber was added to the crude oil spot. The beaker was left for 5 min for the composites to interact with crude oil and form a semisolid paste that could be removed using a magnet. The crude oil amount (Or) removed from the water was determined by gravimetry using Equation (4):

(4) $$\mathrm{Or}=\frac{w_{2}-w_{3}}{w_{1}}$$

where *w*_1_ is the composite weight, *w_2_* is the total weight (beaker with water and crude oil), and *w*_3_ is the system weight after removal (beaker with water and residual crude oil). This method allows obtaining the ratio between the removed crude oil and the composite (g/g).

## 3. Results

**Figure 2:**
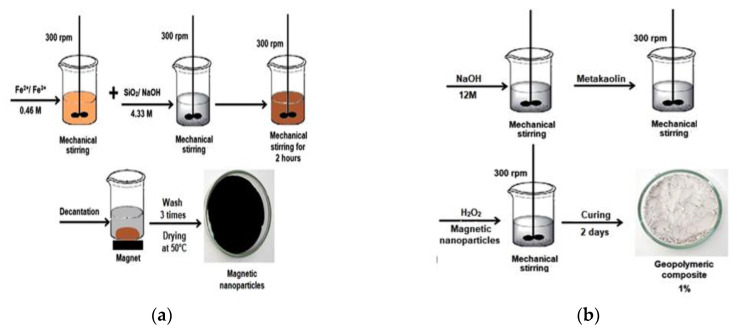
Modification process of magnetic nanoparticles with SiO2 (**a**) and production of geopolymer composites (**b**).

The silica-modified magnetite and geopolymer production are shown in Figure 2a,b, respectively.

**Figure 3:**
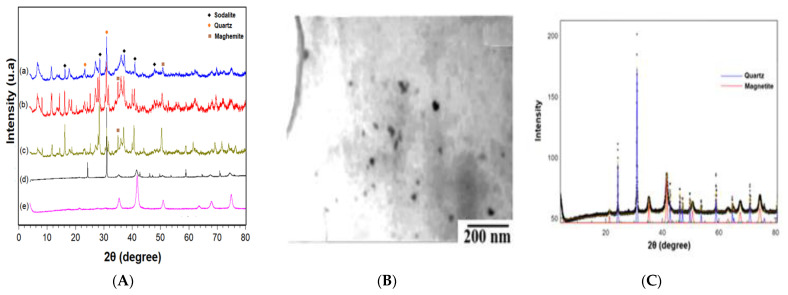
X-Ray diffraction (**A**): (a) geopolymer wt 3%; (b) geopolymer wt 2%; (c) geopolymer wt 1%; (d) magnetic nanoparticles; (e) magnetite. (**B**) TEM of the magnetite and (**C**) Full Profile Search Match quantification quartz and magnetite.

The X-ray diffraction analyses were carried out to identify the crystalline peaks related to geopolymers’ formation with magnetic particles and the modification of the magnetic particles with SiO_2_. Thus, it was possible to identify peaks at 17° angles in the composites, 27.5°, 37°, 41°, and 47° referring to the Sodalite phase and the 24° and 30° peaks of quartz, these being the main formation phases of geopolymers [31]; furthermore, the peaks centered at 35° and 51° identified the presence of magnetite inserted in the geopolymer matrix shows the Figure 3A(a) [32,33,34,35,36].

The magnetite modified with SiO_2_ presented peaks from quartz centered at 24° and 30° besides magnetite peaks. The Full Profile Search Match Software allowed calculating the presence of 82% quartz and 18% magnetite in the sample. These results prove the modification. Moreover, the Scherrer equation was used to define the particle size of the magnetite and silica-modified magnetite. The diameter results calculated at 2θ equal to 35° were equal to 30 nm. This diameter value is in complete agreement with the diameter obtained from TEM, which was equal to (29 ± 3) nm (Figure 3A(b,c)).

**Figure 4:**
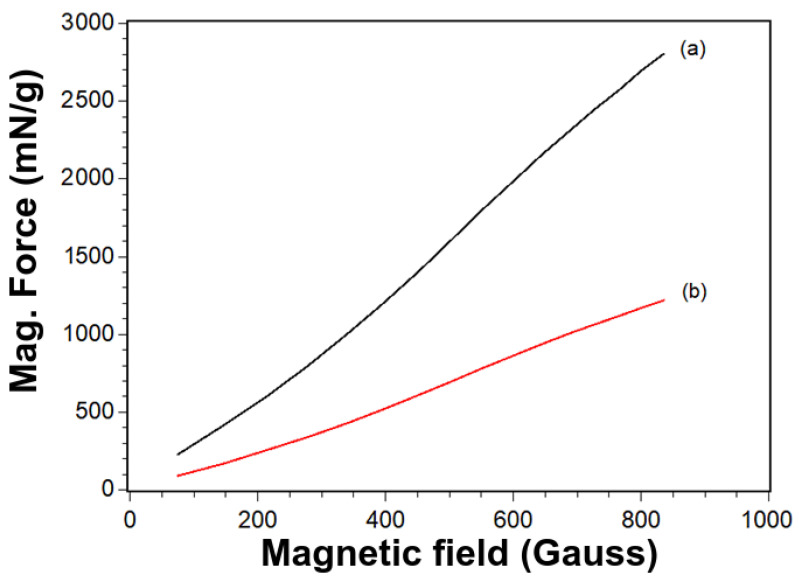
Normalized magnetic force as a function of the magnetic field for magnetite (**a**) and silica-modified (**b**) samples.

The magnetic force tests proved silica’s influence on the magnetite nanoparticles under a magnetic field. The results showed that the magnetite presented a magnetic force of 2804 ± 20 mN/g. In turn, the silica-modified magnetite presented a magnetic force equal to 1218 ± 43 mN/g. Thus, the presence of silica on the magnetite core reduced the magnetic force by 2.3 times. This result is typical in modified magnetic nanoparticles [26]. Besides that, the magnetic response is entirely sufficient to attract the particles during further tests (Figure 4).

**Figure 5:**
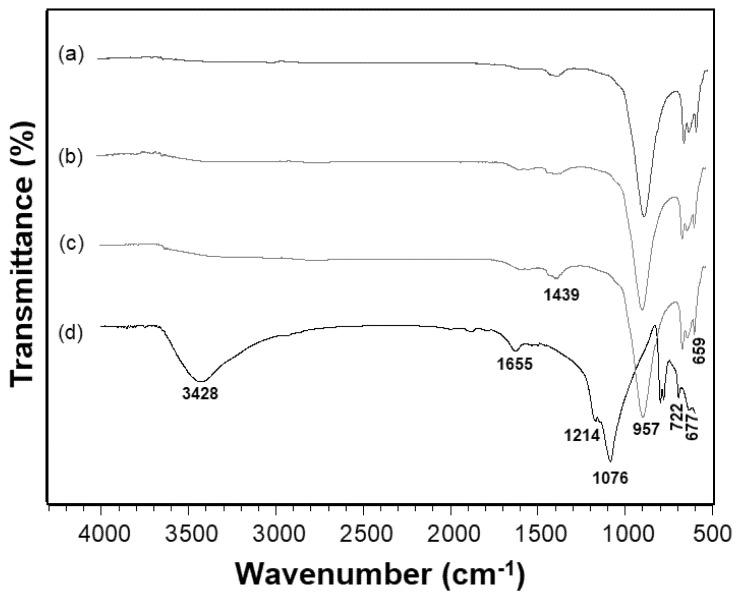
FTIR of the geopolymer filled with 3% (**a**), 2% (**b**), and 1% (**c**) of magnetite and magnetite nanoparticles (**d**).

The FTIR spectra showed bands characteristic of the modification carried out on the geopolymers with the magnetic particles and the modification with SiO_2_ carried out on the magnetite. The composites presented bands centered at 659 cm^−1^ and 722 cm^−1^ from the silicon oxide (Si-O); 677 cm^−1^ from the stretching of the iron oxide (Fe-O); and 957 cm^−1^, which was the more intense band, indicating the asymmetric elongation of the silicon, aluminum and oxygen bond (Si-O-Al). The band at 1439 cm^−1^ is related to carbonates from atmospheric CO_2_ reacting with NaOH from the sample. The stretching vibration of the hydroxyl (OH) is centered at 1626 cm^−1^ (see Figure 5a–c). For the silica-modified magnetite, the band at 677 cm^−1^ is from the iron oxide (Fe-O) stretching, while the one at 1076 cm^−1^ is from the asymmetric stretching of SiO_2_. Finally, the stretching band of ferrous sulfate (SO_4_^2−^) is centered at 1214 cm^−1^, while the ones from the water hydroxyl are centered at 1655 cm^−1^ and 3428 cm^−1^ [37].

**Figure 6:**
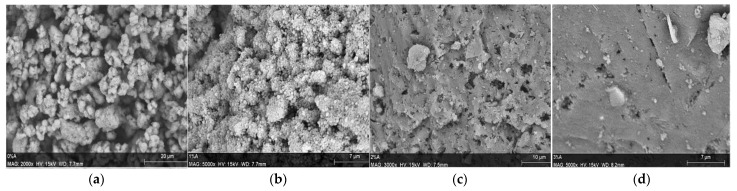
Scanning Electron Microscopy of the geopolymer (**a**) and composites filled with 1 wt% (**b**), 2 wt% (**c**), and 3 wt% (**d**) of the magnetic nanoparticles.

The samples’ SEM micrographs are shown in Figure 6. Scanning Electron Microscopy was performed to verify the geopolymers’ morphology before and after the insertion of the magnetic fillers. Thus, it was possible to identify that the geopolymer without magnetic filler presented a porous structure provided by H_2_O_2_, as shown in Figure 6a. The porosity observed by SEM seems to decrease as the insertion of the magnetic nanofiller in the matrix increases. Moreover, the increasing amount of filler produced agglomerates on the surface leads to the hypothesis that the increasing presence of magnetic nanoparticles produced a material with a more compact structure (see Figure 6b–d). On the contrary, porosity determined by water saturation presented results equal to (28 ± 6)%, (24 ± 6)%, and (26 ± 1)% for the samples filled with 1 wt%, 2 wt%, and 3 wt% of magnetic nanoparticles, respectively. Thus, with 95% of confidence, the porosity values were statistically equal. Besides that, the presence of the micrograins proved that all the tested materials possess a large surface area, helping contaminant sorption.

**Figure 7:**
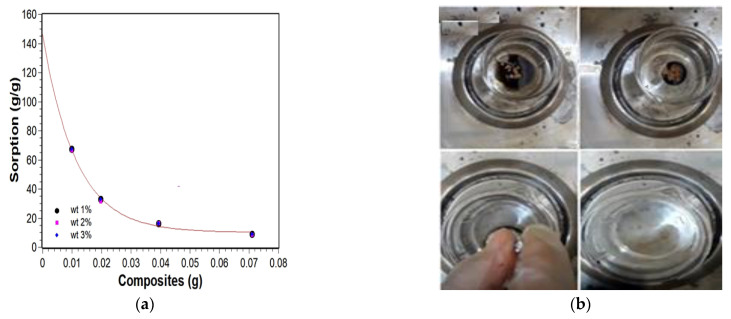
Sorption test geopolymer. (**a**) wt 1%, wt 2%, wt 3%. (**b**) Sorption test.

The sorption tests were performed following the analytical method from the Biopolymers and Sensors Lab [38]. The obtained results are shown in Figure 7. The geopolymer composites were subjected to the sorption process in order to evaluate the material’s ability to retain the contaminant in its structure, which, in this case, is oil. For this, the tests were performed, and the results obtained are shown in Figure 7. Using QtiPlot, graphs were plotted using the function showed in Equation (5):

(5) $${\mathrm{ORC}=\mathrm{ORC}}_{0}+\mathrm{Aexp}\frac{-\left[\mathrm{MNC}\right]}{t}$$

where ORC_0_ is the function’s offset, A is its amplitude, [MNC] is the amount in grams of the magnetic nanocomposites, and *t* is the e-folding time.

Table 1 shows the data obtained in exponential decay. Through this, it was possible generate graphs in which the decay of the curve indicates the sorption capability depending on the amount of material used. Besides that, exponential models allowed inferring the intrinsic oil removal capability (IORC) of the prepared samples. These calculations were performed by the extrapolation of the magnetic nanocomposites amount to zero, shown in Equation (6):

(6) $$\lim_{\mathrm{MNC}\to 0}\mathrm{ORC}\left(\mathrm{MNC}\right)=\mathrm{IORC}$$

Data proved that, the smaller the amount of geopolymer in the oil slick, the greater the sorption capability of this material. The activating agents present in the geopolymer structure allow for lower steric hindrance, which facilitates the adsorption of carboxylated and olefinic compounds present in petroleum by the geopolymer matrix [39]. In this case, geopolymer-based materials can remove (67 ± 1) g of oil per 1 g of geopolymer. Moreover, the amount of filler in the matrix did not influence the sorption capability of the material. In turn, the composites’ intrinsic oil removal capability (ORC0) values are statistically equal because of the calculated porosity values, which are also statistically equal. The calculated ORC0 values are shown in Table 1, and they are, on average, equal to (148 ± 6) g/g.

As shown in a study from our group, performed by Figueiredo et al. (2019), the higher the number of magnetic particles, the higher the speed of oil removal from water by the higher magnetic particle/magnet interaction. The former relationship provides a strongly connected network, which presents voids that can trap petroleum, and the oil removal efficiency increases due to the pores of the geopolymer matrices [23].

## 4. Discussion

These results prove the geopolymers’ potential to contribute to environmental remediation. Moreover, geopolymers have proved to be more efficient in oil removal applications than other materials, such as the zinc-doped magnetic particles of cobalt ferrite, which has an oil removal capability of approximately 14 g/g of the sorber [40]. Polyurethane resins modified with magnetic particles, on the other hand, can remove 10 g of oil from the water per gram of the composite [41]. Another composite prepared with magnetic wood sawdust presented an oil removal efficiency of 28 g/g [42]. In turn, a magnetic nanostructured graphene composite was produced for oil removal, and the results showed that the oil removal capability was equal to 53 g/g [43]. In this sense, the geopolymers here presented can remove at least 67 g/g of the oil from the water, constituting an easy-to-produce, cheap, and promising material that can contribute to the environmental recovery of oil impacted areas.

## 5. Conclusions

The XRD and FTIR techniques showed the production of geopolymers modified by magnetic nanoparticles and confirmed the magnetic nanoparticles’ modification with SiO_2_. TEM results are in complete agreement with the XRD results, proving the nanoscopic nature of the magnetic nanoparticles prepared in this study. The analyses made in Scanning Electron Microscopy showed the formation of pores in the materials. These pores are fundamental to the extraordinary oil sorption capacity presented by these materials. Sorption tests have shown that the materials produced have an average intrinsic oil removal capability of around 150 g/g. The number of nanoparticles used in the nanocomposites’ preparation did not influence the intrinsic oil removal capability, demonstrating that the geopolymer material is primarily responsible for differentiated oil removal capacity from water. This extraordinary response, coupled with the low cost of the geopolymer material and its ease of preparation, allows us to consider that geopolymer matrices are up-and-coming candidates for environmental recovery applications involving oil spills.

## Figures and Tables

**Table 1:** Data obtained in exponential decay.

Magnetite (%)	A	t	ORC_0_	R2	IORC (g/g)
1	(1.35 ± 0.21) × 10^2^	(1.12 ± 0.02) × 10^−2^	(1.05 ± 0.26) × 10^1^	0.996	147 ± 24
2	(1.40 ± 0.27) × 10^2^	(1.06 ± 0.02) × 10^−2^	(1.04 ± 0.30) × 10^1^	0.994	150 ± 30
3	(1.36 ± 0.2) × 10^2^	(1.12 ± 0.02) × 10^−2^	(1.02 ± 0.25) × 10^1^	0.996	146 ± 23

## Data Availability

Data are contained within the article.

## References

[1] Beyer J., Trannum H.C., Bakke T., Hodson P.V., Collier T.K. (2016). Environmental Effects of the Deepwater Horizon Oil Spill: A Review. Mar. Pollut. Bull..

[2] Araújo M.E.D., Ramalho C.W.N., Melo P.W.D. (2020). Artisanal Fishers, Consumers and the Environment: Immediate Consequences of the Oil Spill in Pernambuco, Northeast Brazil. Cad. Saúde Pública.

[3] Bullock R.J., Perkins R.A., Aggarwal S. (2019). N-Situ Burning with Chemical Herders for Arctic Oil Spill Response: Meta-Analysis and Review. Sci. Total Environ..

[4] Siyal A.A., Shamsuddin M.R., Khan M.I., Rabat N.E., Zulfiqar M., Man Z., Azizli K.A. (2018). A Review on Geopolymers as Emerging Materials for the Adsorption of Heavy Metals and Dyes. Ournal. Environ. Manag..

[5] EL-ESWED (2018). BASSAM I Solidification Versus Adsorption for Immobilization of Pollutants in Geopolymeric Materials: A Review. Solidification.

[6] Yuan F., Wei S., Zhichao L., Qiuyi W., Yihan Z., Yanjun L. (2021). An industrial feasible and sustainable method for preparing fiberized bamboo-derived magnetic biomass carbon. J. Mater. Sci. Mater. Electron..

[7] Yuan C., Lou Z., Wang W., Yang L., Li Y. (2019). Synthesis of Fe_3_C@ C from Pyrolysis of Fe_3_O_4_-Lignin clusters and its application for quick and sensitive detection of PrPSc through a sandwich SPR detection assay. Int. J. Mol. Sci..

[8] Yang L., Deng T., Jia Z., Zhou X., Lv H., Zhu Y., Yang Z. (2021). Hierarchical porous hollow graphitized carbon@ MoS2 with wideband EM dissipation capability. J. Mater. Sci. Technol..

[9] Maranhão F.S., de Souza Junior F.G., Filho S.T., de Oliveira Athayde B.H., de Carvalho F.F., Lino A., Malm O. (2021). Magnetic Porous Geopolymer: A Cheaper and Efficient Environmental Tool for Heavy Metal Sorption. Macromol. Symp..

[10] Maranhão F.D.S., Almeida T.M., de Souza F.G., Batista D., de Carvalho F.F., Pal K., Thomas S. (2021). Geopolymer Microparticles as Up‐and‐Coming H_2_S Sorbers. Macromol. Symp..

[11] Maranhão F.D.S., Batista D., Motta A.G., de Carvalho F.F., de Almeida T.M., Das D.B., Junior F.G. (2021). Study of the Geopolymer Sorption Capacity Exposed To Hydrogen Sulfide (H_2_S). Multidiscip. Sci. Adv. Technol..

[12] Ge Y., Cui X., Kong Y., Li Z., He Y., Zhou Q. (2015). Porous Geopolymeric Spheres for Removal of Cu (II) from Aqueous Solution: Synthesis and Evaluation. J. Hazard. Mater..

[13] Davidovits J. (2008). Joseph Geopolymer Chemistry and Applications. Geopolymer Inst..

[14] Ferreira L.P., Moreira A.N., Delazare T., Oliveira G.E., Souza F.G. (2012). Petroleum Absorbers Based on CNSL, Furfural and Lignin The Effect of the Chemical Similarity on the Interactions among Petroleum and Bioresins. Macromol. Symp..

[15] Elias E., Costa R., Marques F., Oliveira G., Guo Q., Thomas S., Souza F.G. (2015). Oil-Spill Cleanup: The Influence of Acetylated Curaua Fibers on the Oil-Removal Capability of Magnetic Composites. J. Appl. Polym. Sci..

[16] Silva J.C., Oliveira G.E., Toledo Filho R.D., Souza F.G. (2018). Oil Spill Clean-Up Tool Based on Castor Oil and Coffee Grounds Magnetic Resins. Macromol. Symp..

[17] Marques F.D., Souza F.G., Oliveira G.E. (2016). Oil Sorbers Based on Renewable Sources and Coffee Grounds. J. Appl. Polym. Sci..

[18] Caetano R.M.J., Bedor P.B.A., de Jesus E.F.O., Leite S.G.F., Souza F.G. (2018). Oil Biodegradation Systems Based on γ Irradiated Poly (Butylene Succinate). Macromol. Symp..

[19] Grance E.G.O., Souza F.G., Varela A., Pereira E.D., Oliveira G.E., Rodrigues C.H.M. (2012). New Petroleum Absorbers Based on Lignin-CNSL-formol Magnetic Nanocomposites. J. Appl. Polym. Sci..

[20] Oliveira G.E., Souza F.G., Lopes M.C. (2012). Magnetic Biofoams Based on Polyurethane Applied in Oil Spill Cleanup Processes Chapter 23. Natural Polymers, Biopolymers, Biomaterials, and Their Composites, Blends, and IPNs CRC Press Book.

[21] Varela A., Lopes M.C., Delazare T., Oliveira G.E., Souza F.G. (2012). Magnetic and green resins useful to oil spill cleanup. Oil: Production, Consumption and Environmental Impact.

[22] Costa R.M.D., Hungerbühler G., Saraiva T., De Jong G., Moraes R.S., Furtado E.G., Silva F.M., Oliveira G.E., Ferreira L.S., Souza F.G. (2017). Green Polyurethane Synthesis by Emulsion Technique: A Magnetic Composite for Oil Spill Removal. Polímeros.

[23] Figueiredo A.S., Icart L.P., Marques F.D., Fernandes E.R., Ferreira L.P., Oliveira G.E., Souza F.G. (2019). Extrinsically Magnetic Poly(Butylene Succinate): An up-and-Coming Petroleum Cleanup Tool. Sci. Total Environ..

[24] Lopes M.C., Marques F., Souza F.G., Oliveira G.E. (2018). Experimental Design Optimization of Castor Oil, Phthalic Anhydride, and Glycerin Magnetic Nanocomposites Useful as Oil Spill Cleanup Tool. Macromol. Symp..

[25] Souza F.G., Oliveira G.E., Lopes M.C. (2012). Environmental Recovery by Magnetic Nanocomposites Based on Castor Oil Chapter 22. Natural Polymers, Biopolymers, Biomaterials, and Their Composites, Blends, and IPNs-CRC Press Book.

[26] Souza F.G., Marins J.A., Rodrigues C.H.M., Pinto J.C. (2010). A Magnetic Composite for Cleaning of Oil Spills on Water. Macromol. Mater. Eng..

[27] Muniz F.T.L., Miranda M.R., Morilla dos Santos C., Sasaki J.M. (2016). The Scherrer Equation and the Dynamical Theory of X-Ray Diffraction. Acta Crystallogr. Sect. Found. Adv..

[28] Cathedralcollege “How to Calculate Porosity|Solutions|September 2021”. https://pt.cathedralcollege.org/calcular-la-porosidad-4755.

[29] Rodrigues V.D.S., Bezerra F.M., Sousa G.G.D., Fiusa J.N., Leite K.N., Viana T.V.D.A. (2019). Yield of maize crop irrigated with saline waters. Rev. Bras. De Eng. Agrícola E Ambient..

[30] Wang X., Li M., Shen Y., Yang Y., Feng H., Li J. (2019). Facile preparation of loess-coated membranes for multifunctional surfactant-stabilized oil-in-water emulsion separation. Green Chem..

[31] Zheng Z., Ma X., Zhang Z., Li Y. (2019). In-Situ Transition of Amorphous Gels to Na-P1 Zeolite in Geopolymer: Mechanical and Adsorption Properties. Constr. Build. Mater..

[32] Kloster G.A., Muraca D., Meiorin C., Pirota K., Marcovich N.E., Mosiewicki M.A. (2015). Magnetic Characterization of Chitosan–Magnetite Nanocomposite Films. Eur. Polym. J..

[33] Soto G.D., Meiorin C., Actis D.G., Mendoza Zélis P., Moscoso Londoño O., Muraca D., Mosiewicki M.A., Marcovich N.E. (2018). Magnetic Nanocomposites Based on Shape Memory Polyurethanes. Eur. Polym. J..

[34] Meiorin C., Actis D.G., Montoro F.E., Londoño O.M., Aranguren M.I., Muraca D., Zélis P.M., Knobel M., Mosiewicki M.A. (2018). Magnetic Remote Activation of Shape Recovery in Nanocomposites Based on Tung Oil and Styrene. Phys. Status Solidi A.

[35] Meiorin C., Londoño O.M., Muraca D., Socolovsky L.M., Pirota K.R., Aranguren M.I., Knobel M., Mosiewicki M.A. (2016). Magnetism and Structure of Nanocomposites Made from Magnetite and Vegetable Oil Based Polymeric Matrices. Mater. Chem. Phys..

[36] Soto G.D., Meiorin C., Actis D., Mendoza Zélis P., Mosiewicki M.A., Marcovich N.E. (2018). Nanocomposites with Shape Memory Behavior Based on a Segmented Polyurethane and Magnetic Nanostructures. Polym. Test..

[37] Król M., Rożek P., Chlebda D., Mozgawa W. (2019). ATR/FT-IR Studies of Zeolite Formation during Alkali-Activation of Metakaolin. Solid State Sci..

[38] Lee S. (2020). Beneficial Use of MIBC in Metakaolin-Based Geopolymers to Improve Flowability and Compressive Strength. Materials.

[39] Marinho V., Lima N., Neves M.A., Souza F. (2021). Petroleum Sorbers Based on Renewable Alkyd Resin and Lignin. Macromol. Symp..

[40] Maleki A., Hajizadeh Z., Sharifi V., Emdadi Z. (2019). A Green, Porous and Eco-Friendly Magnetic Geopolymer Adsorbent for Heavy Metals Removal from Aqueous Solutions. J. Clean. Prod..

[41] Amar I.A., Alshibani Z.M., AbdulQadir M.A., Abdalsamed I.A., Altohami F. (2019). A Oil Spill Removal from Water by Absorption on Zinc-Doped Cobalt Ferrite Magnetic Nanoparticles. Adv. J. Chem.-Sect. Theor. Eng. Appl. Chem..

[42] Soliman E.M., Ahmed S.A., Fadl A. (2020). A Adsorptive Removal of Oil Spill from Sea Water Surface Using Magnetic Wood Sawdust as a Novel Nano-Composite Synthesized via Microwave Approach. J. Environ. Health Sci. Eng..

[43] Ramirez Leyva J.H., Hethnawi A., Vitale G., Nassar N. (2018). N Magnetic Nanostructured White Graphene for Oil Spill and Water Cleaning. Ind. Eng. Chem. Res..

